# Supplementary material for: Neutrophil as a Carrier for Cancer Nanotherapeutics: A Comparative Study of Liposome, PLGA, and Magnetic Nanoparticles Delivery to Tumors
Source: Pharmaceuticals (Basel). 2023 Nov 6;16(11):1564. doi: 10.3390/ph16111564 (PMC10674452; doi:10.3390/ph16111564)
Supplement: Supplementary file 1 [file pharmaceuticals-16-01564-s001.zip › pharmaceuticals-2657987-supplementary.pdf]

Supplementary Information

Neutrophil as a carrier for cancer nanotherapeutics: a comparative study of liposome, PLGA, and magnetic nanoparticles delivery to tumors

Anastasiia S. Garanina [1,*], Daniil A. Vishnevskiy [2,3], Anastasia A. Chernysheva [3], Marat P. Valikhov [2,3], Julia A. Malinovskaya [4], Polina A. Lazareva [2], Alevtina S. Semkina [2,3], Maxim A. Abakumov [1,2], Victor A. Naumenko [3]

[1] Laboratory of Biomedical Nanomaterials, National University of Science and Technology «MISIS», 119049 Moscow, Russia; abakumov1988@gmail.com

[2] Department of Medical Nanobiotechnology, N.I. Pirogov Russian National Research Medical University, 117997 Moscow, Russia; vishnevskiy.daniil.andreevich@gmail.com (D.A.V.); marat.valikhov@gmail.com (M.P.V.); polizara604@gmail.com (P.A.L.); alevtina.semkina@gmail.com (A.S.S.)

[3] V. Serbsky National Medical Research Center for Psychiatry and Narcology, 119034 Moscow, Russia; aachernysheva512@gmail.com (A.A.C.); naumenko.vict@gmail.com (V.A.N.)

[4] D. Mendeleev University of Chemical Technology of Russia, 125047 Moscow, Russia; j.malinowskaya@gmail.com

* Correspondence: anastasiacit@gmail.com

Results

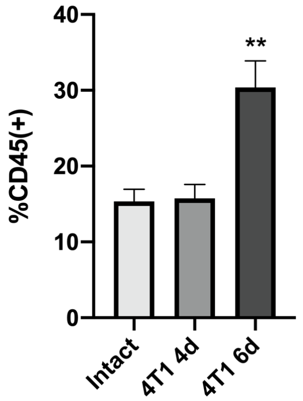

Figure S1. Neutrophil content in the blood of 4T1-bearing BALB/c mice on the 4th and 6th days after tumor cells implantation compared with intact animals, flow cytometry. Results are presented as mean ± SEM. ** $p < 0.01$ (One-way ANOVA followed by Tukey's multiple comparison test). The percentages of CD11b+Ly6G+ positive cells (neutrophils) relative to CD45+ cells (leukocytes) are presented.

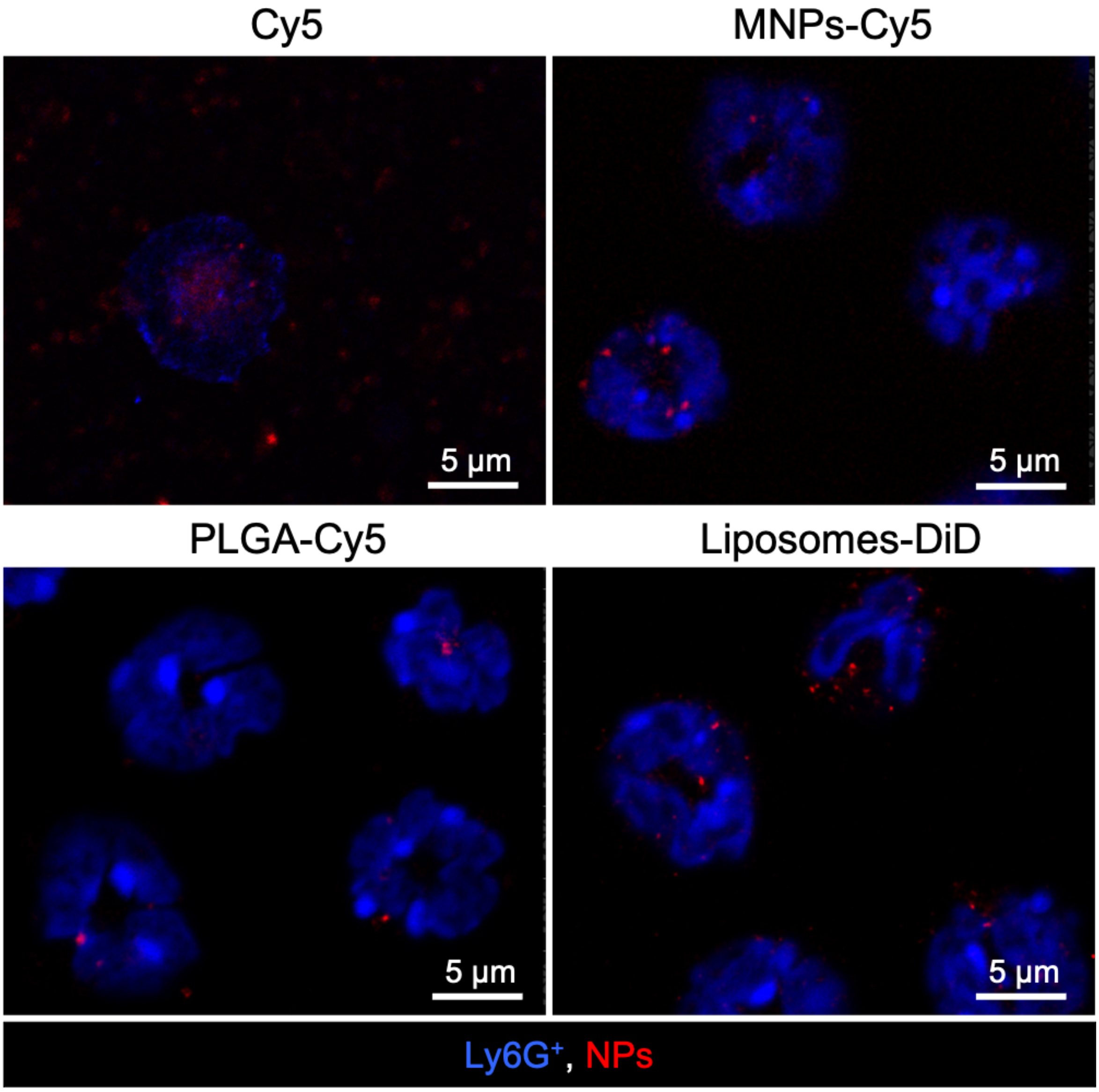

Figure S2. Nanoparticle accumulation inside neutrophils, isolated from the blood of 4T1-bearing mice, after 1 hour of co-incubation, confocal microscopy. Ly6G+ – blue, NPs (MNPs-Cy5, PLGA-Cy5 or liposomes-DiD) – red.

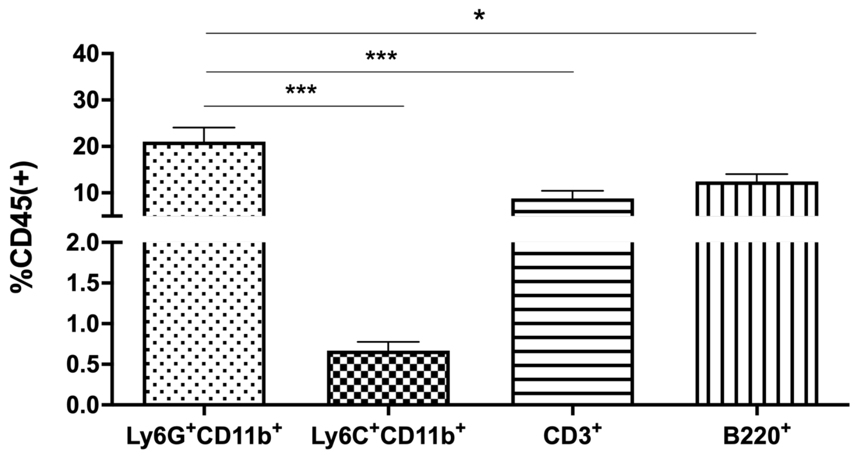

Figure S3. Content of various cells subpopulations in the blood of 4T1-bearing BALB/c mice on the 6th day after tumor cells implantation, flow cytometry. Results are presented as mean ± SEM. * $p < 0.05$, *** $p < 0.001$ (One-way ANOVA followed by Tukey's multiple comparison test). The percentages of Ly6G$^+$CD11b$^+$ (neutrophils), Ly6C$^+$CD11b$^+$ (monocytes), CD3$^+$ (T-lymphocytes), and B220$^+$ (B-lymphocytes) positive cells relative to CD45$^+$ cells (leukocytes) are presented.

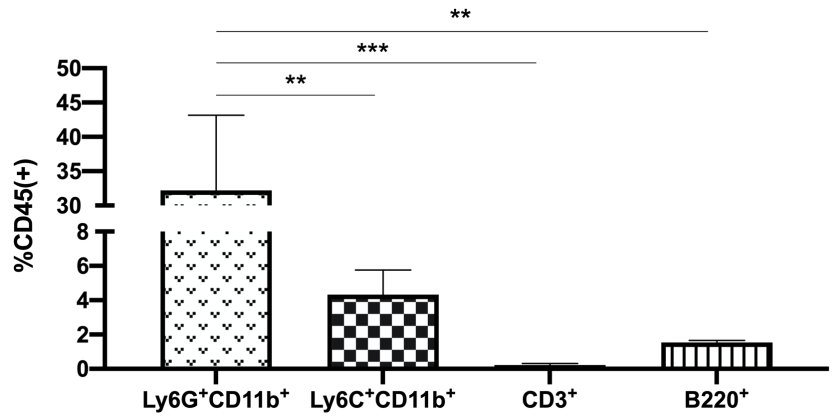

Figure S4. Content of various cells subpopulations in the tumor microenvironment of 4T1-bearing BALB/c mice on the 6th day after tumor cells implantation, flow cytometry. Results are presented as mean ± SEM. ** $p < 0.01$, *** $p < 0.001$ (One-way ANOVA followed by Tukey's multiple comparison test). The percentages of Ly6G$^+$CD11b$^+$ (neutrophils), Ly6C$^+$CD11b$^+$ (monocytes), CD3$^+$ (T-lymphocytes), and B220$^+$ (B-lymphocytes) positive cells relative to CD45$^+$ cells (leukocytes) are presented.

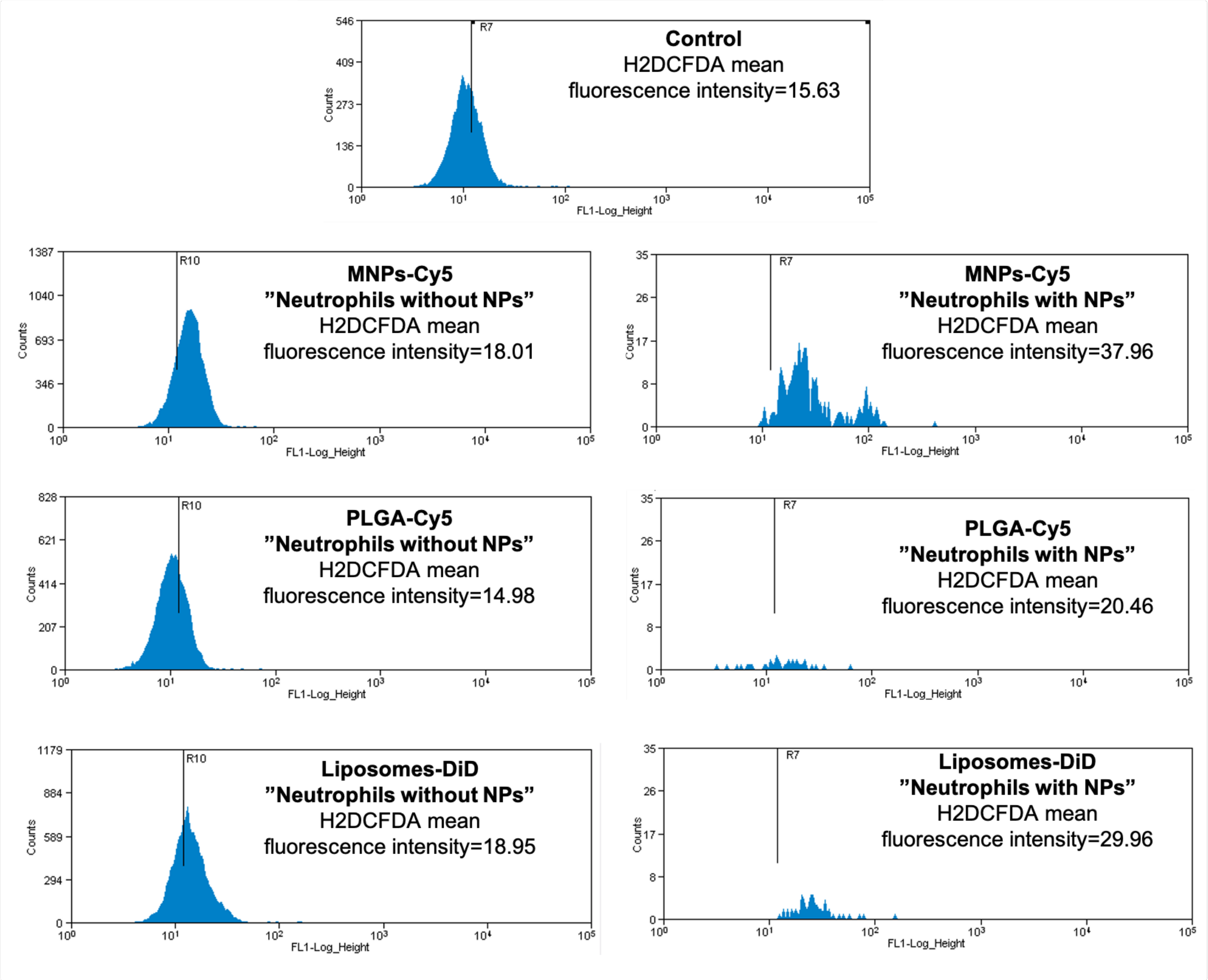

Figure S5. ROS production by neutrophils 30 min after NPs i.v. injection, flow cytometry. Representative histograms of H2DCFDA dye mean fluorescence intensity in neutrophils that have interacted with NPs and not (data of one sample for each type of NPs are presented).

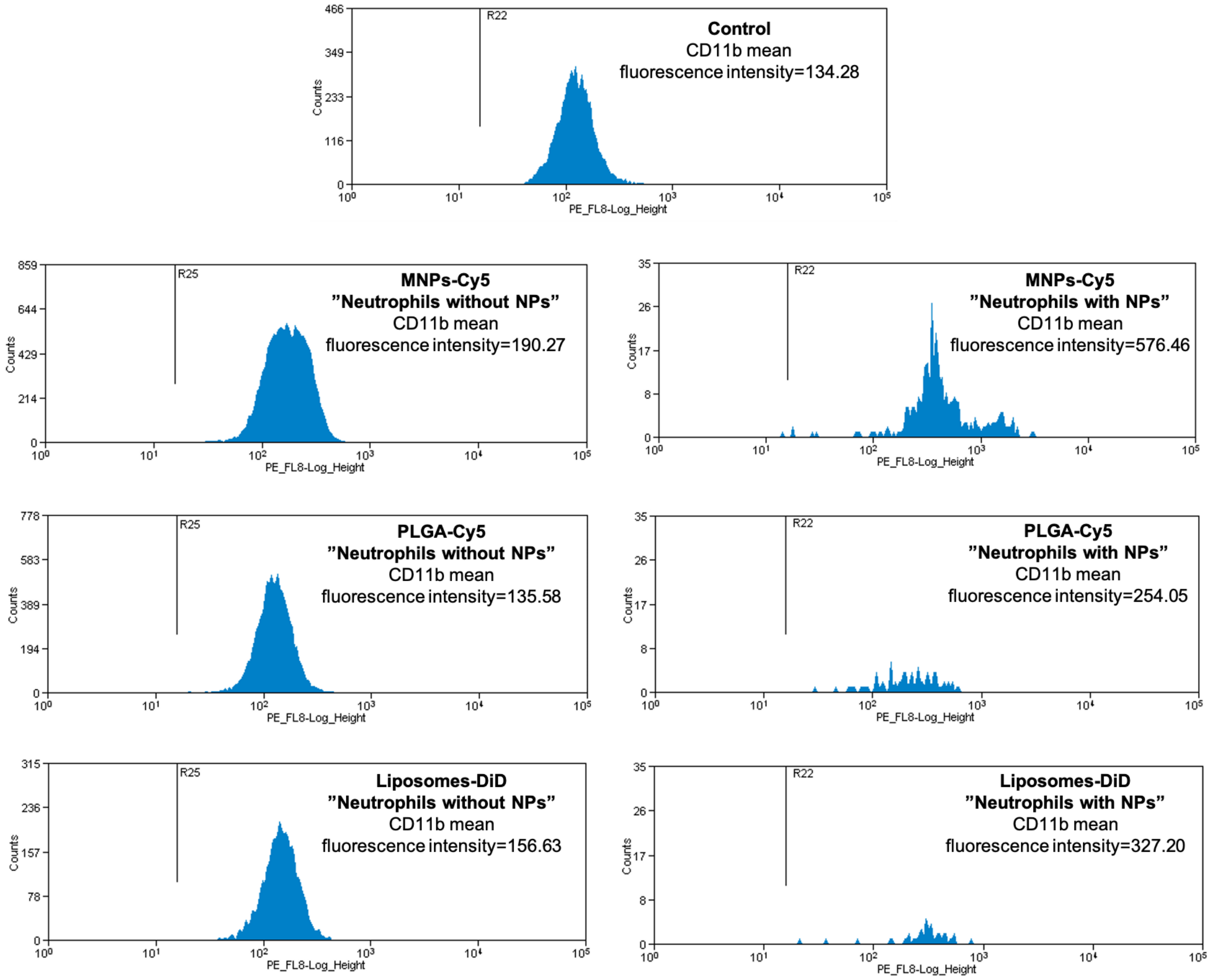

Figure S6. CD11b expression by neutrophils 30 min after NPs i.v. injection, flow cytometry. Representative histograms of CD11b mean fluorescence intensity in neutrophils that have interacted with NPs and not (data of one sample for each type of NPs are presented).

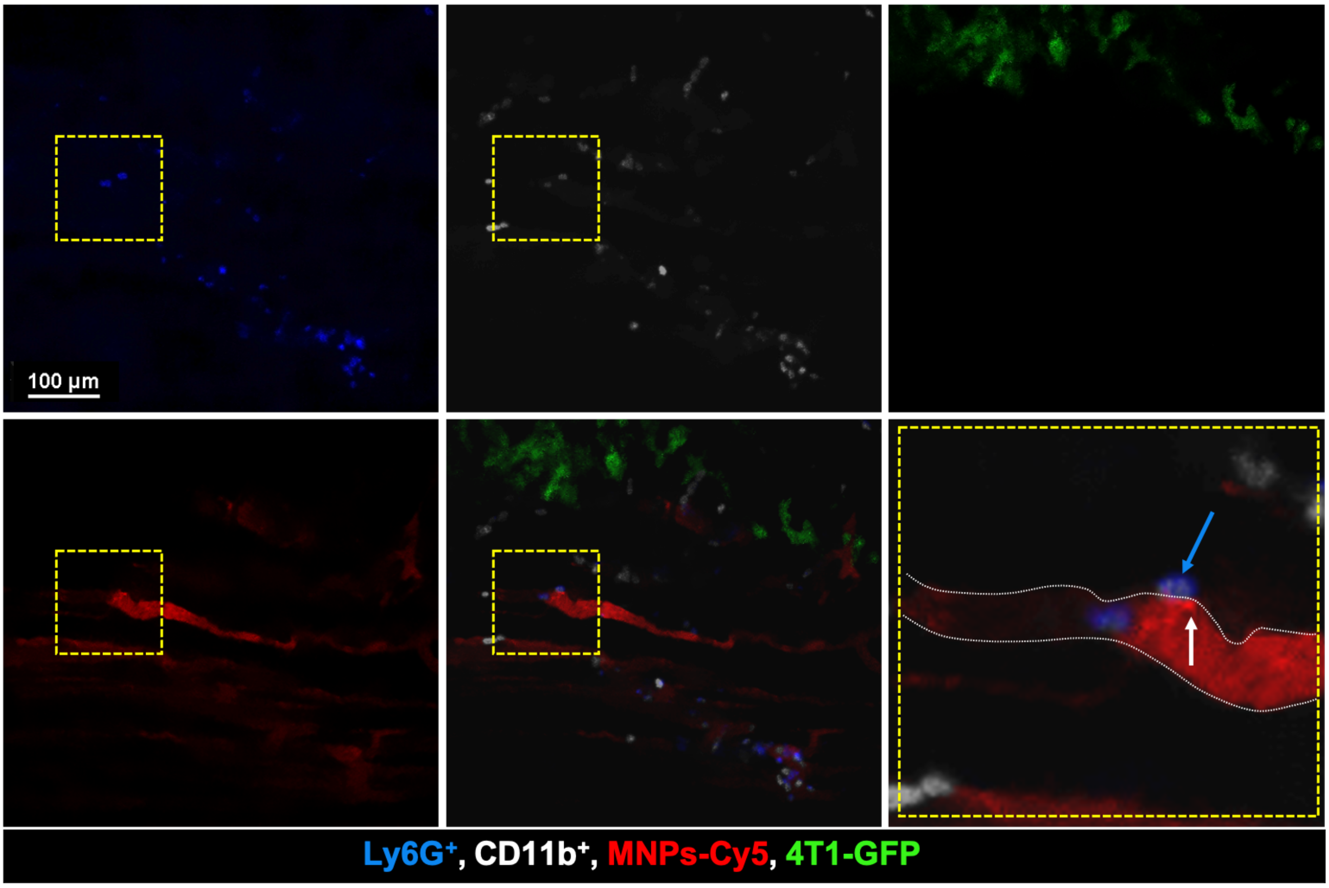

Figure S7. MNPs-Cy5 behavior in tumor vessels 1 hour after i.v. administration to 4T1-GFP tumor-bearing mice, IVM. Blue arrow shows the neutrophil (CD11b+Ly6G+) extravasated from the blood vessel with NPs conglomerate (white arrow). White dotted lines indicate the vessel wall, yellow dotted square – the magnified area of interest.

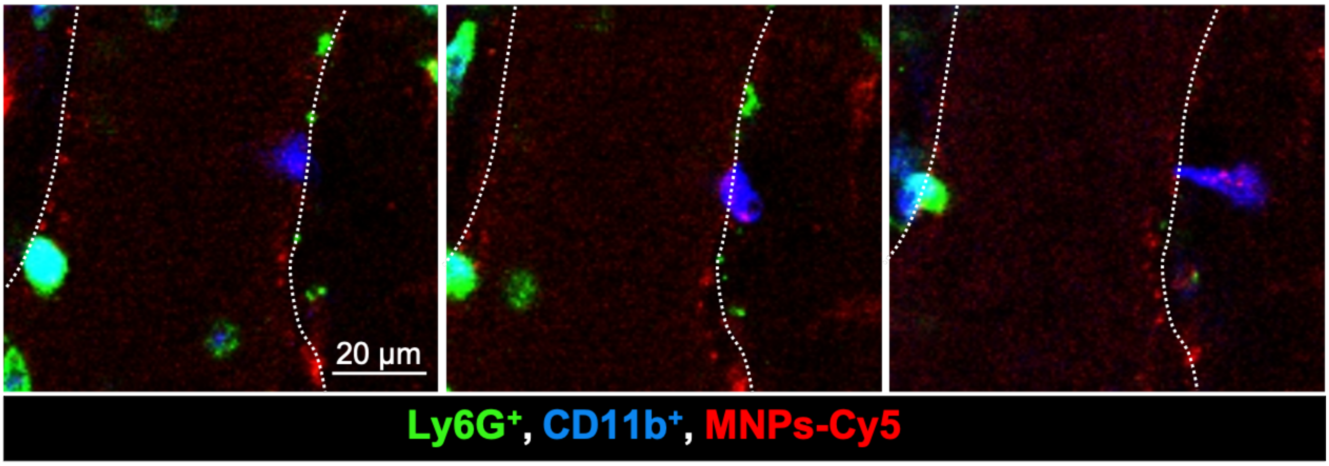

Figure S8. MNPs-Cy5 behavior in tumor vessels 1 hour after i.v. administration to 4T1 tumor-bearing mice, IVM. Monocyte (Ly6C+)-based NPs extravasation, the dotted line represents the vessel wall.

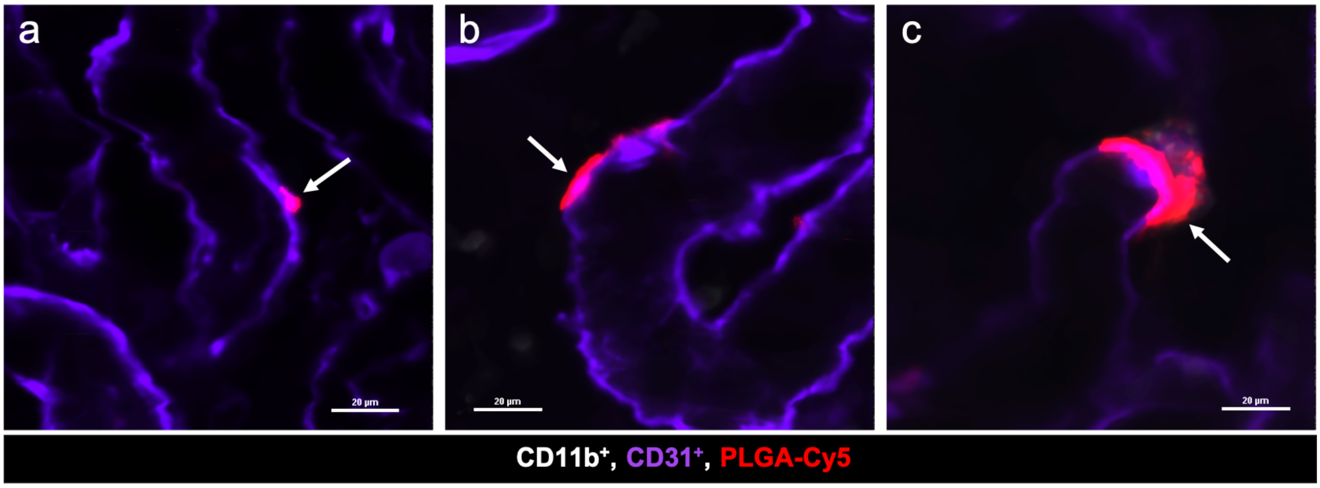

Figure S9. PLGA-Cy5 clusters (shown by arrows) localization relative to the vessel endothelium after i.v. administration to 4T1-GFP tumor-bearing mice, IVM: (**a**) pinpointed cluster; (**b**) elongated cluster; (**c**) cluster "hugging" the vessel.

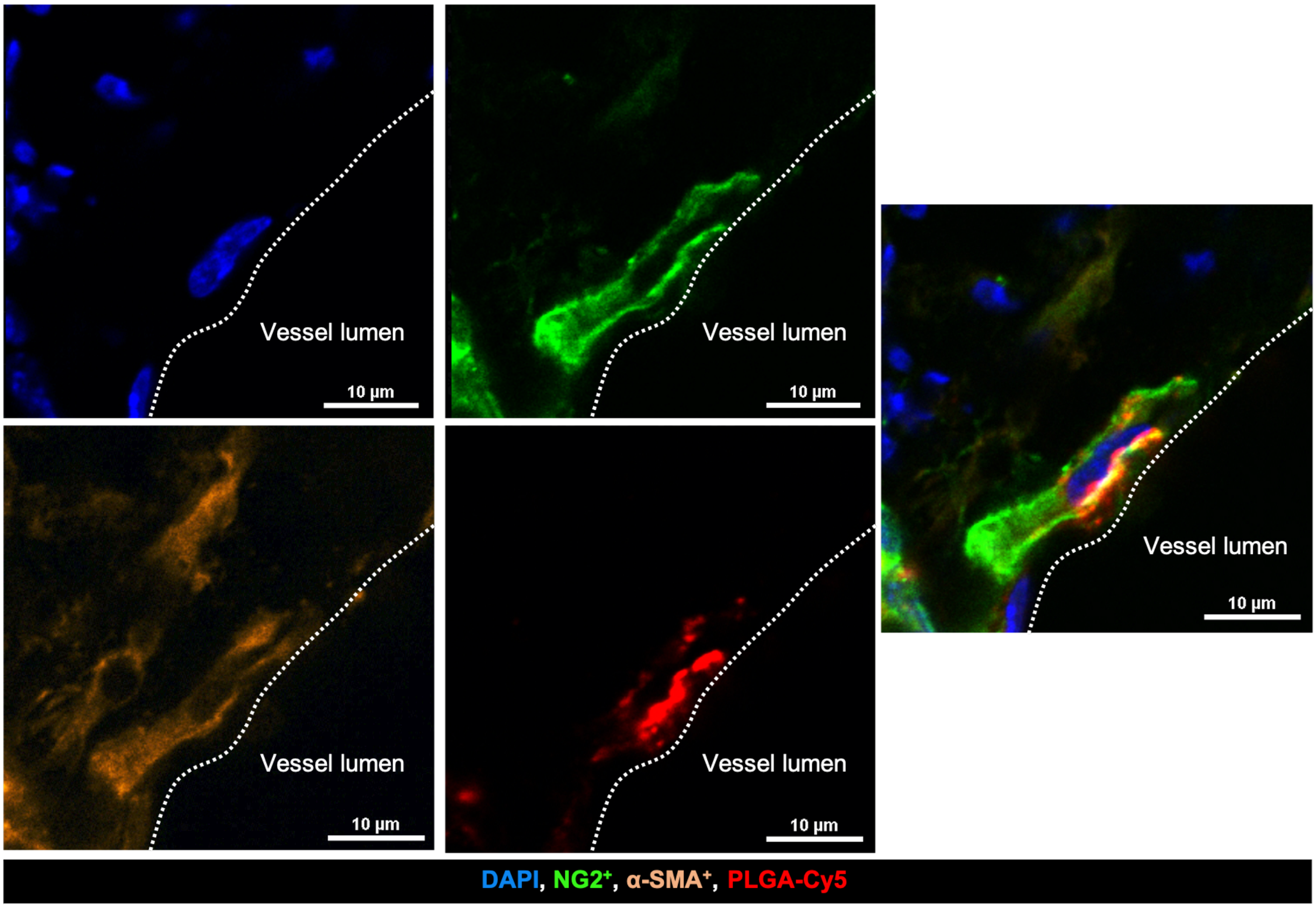

Figure S10. PLGA-Cy5 interaction with pericyte after i.v. administration to 4T1 tumor-bearing mice, immunohistochemistry, confocal microscopy. White dotted line indicates the vessel wall.

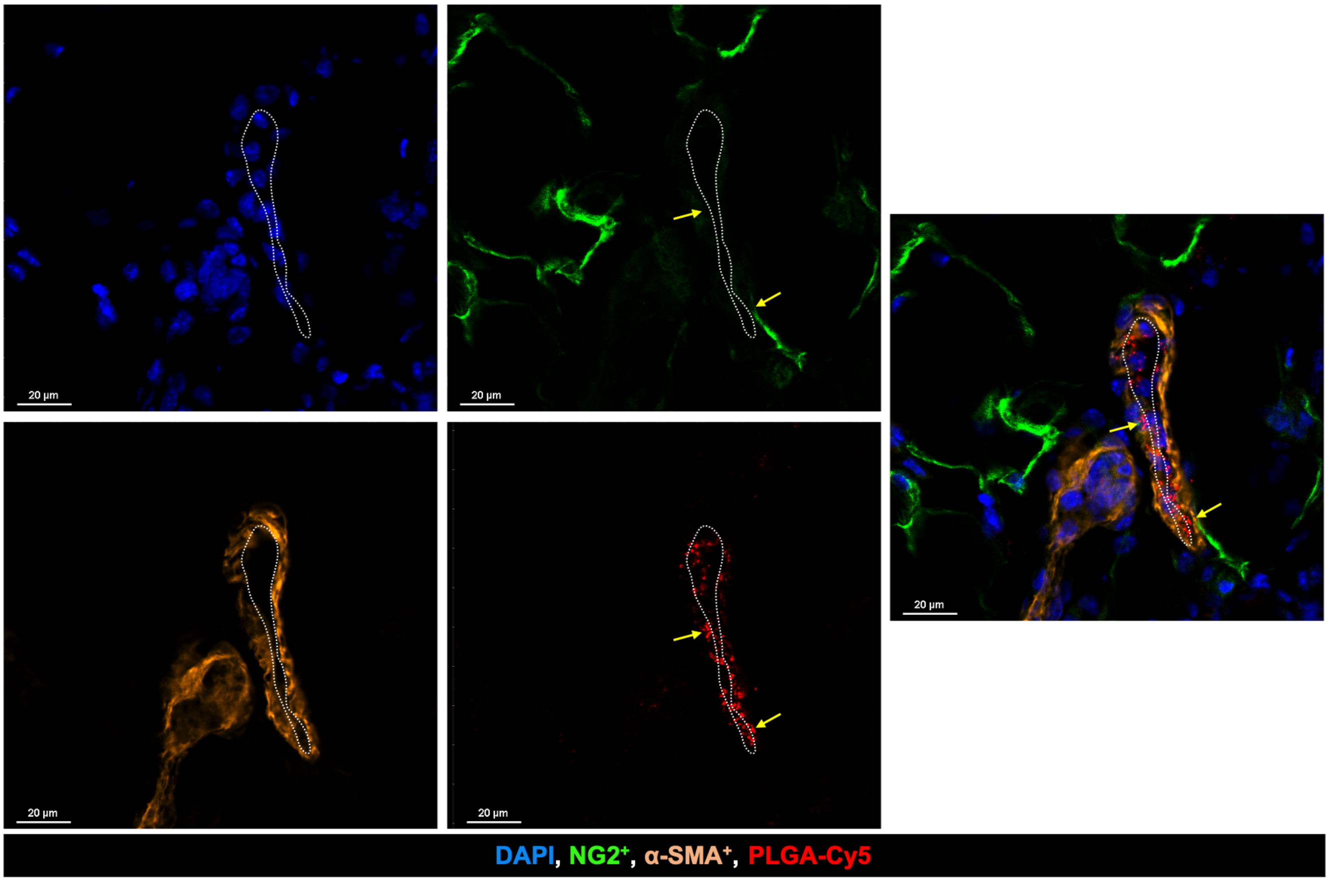

Figure S11. PLGA-Cy5 clusters formation independently of pericytes after i.v. administration to 4T1 tumor-bearing mice, immunohistochemistry, confocal microscopy. Arrows show NPs clusters outside the pericytes. White dotted line indicates the vessel wall.

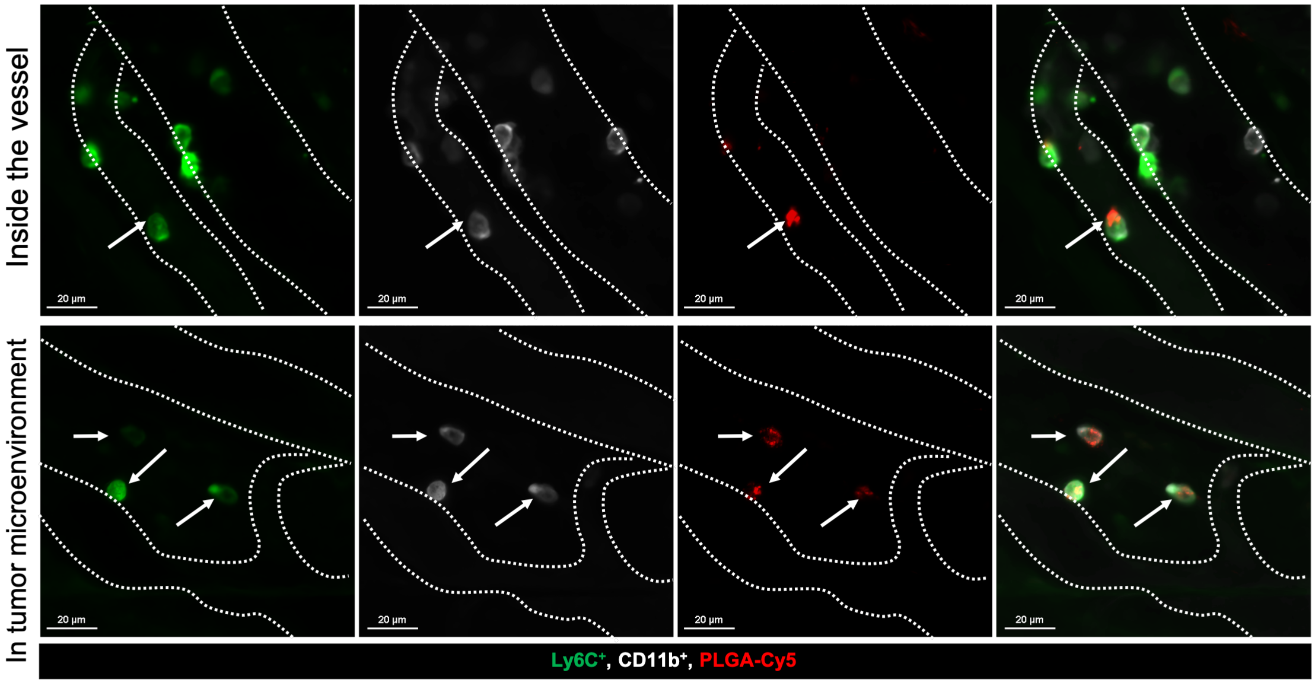

Figure S12. PLGA-Cy5 behavior in tumor vessels 1 hour after i.v. administration to 4T1 tumor-bearing mice, intravital microscopy. Arrows show the monocytes ($CD11b^{+}Ly6C^{+}$) inside the vessel or in tumor microenvironment with accumulated NPs. White dotted lines indicate the vessel walls.

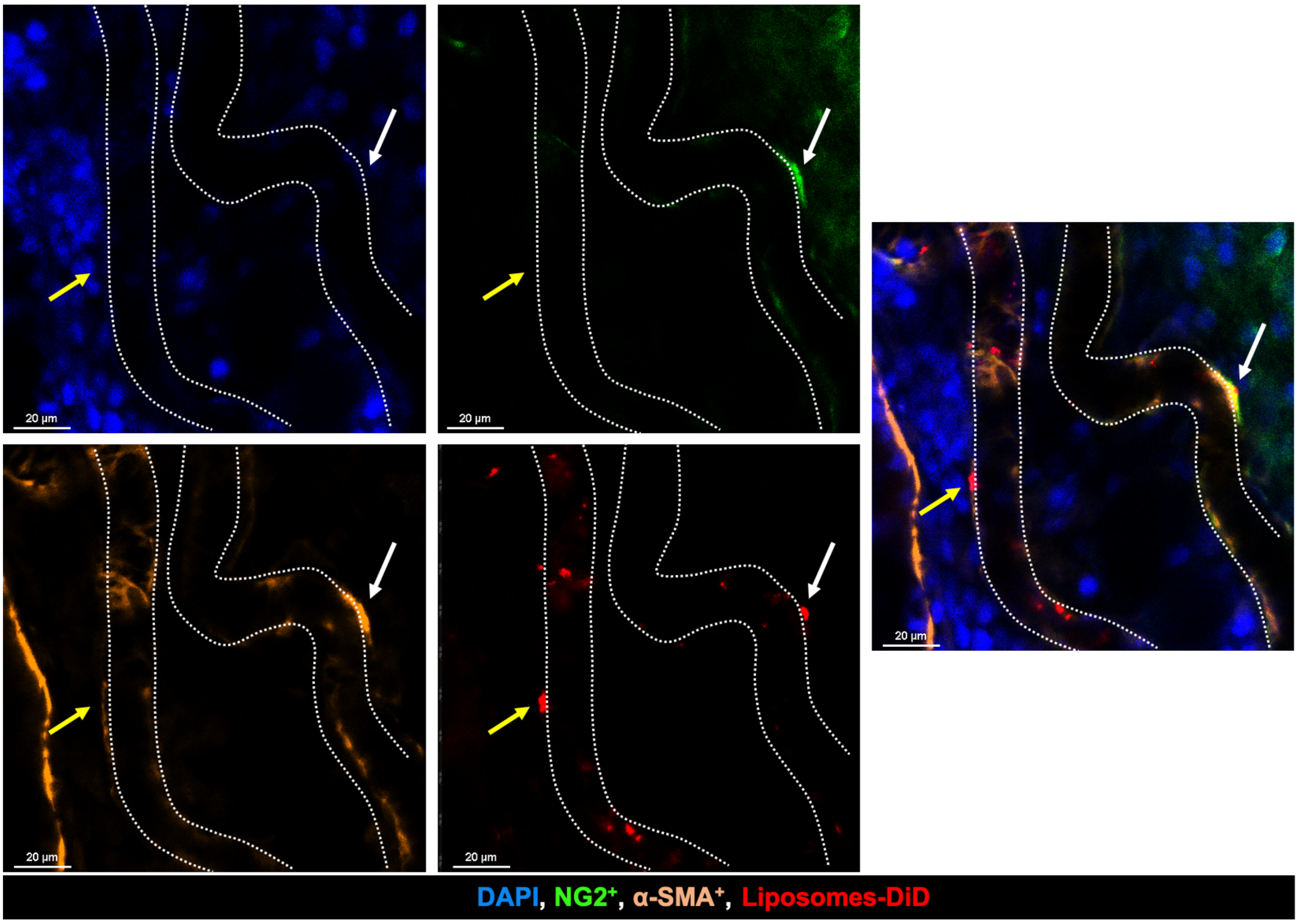

Figure S13. Liposomes-DiD interaction with pericytes after i.v. administration to 4T1 tumor-bearing mice, wholemount staining, confocal microscopy. White arrow shows pericyte with NPs inside, yellow arrow – NPs microleakage independently of pericyte. White dotted lines indicate the vessel walls.

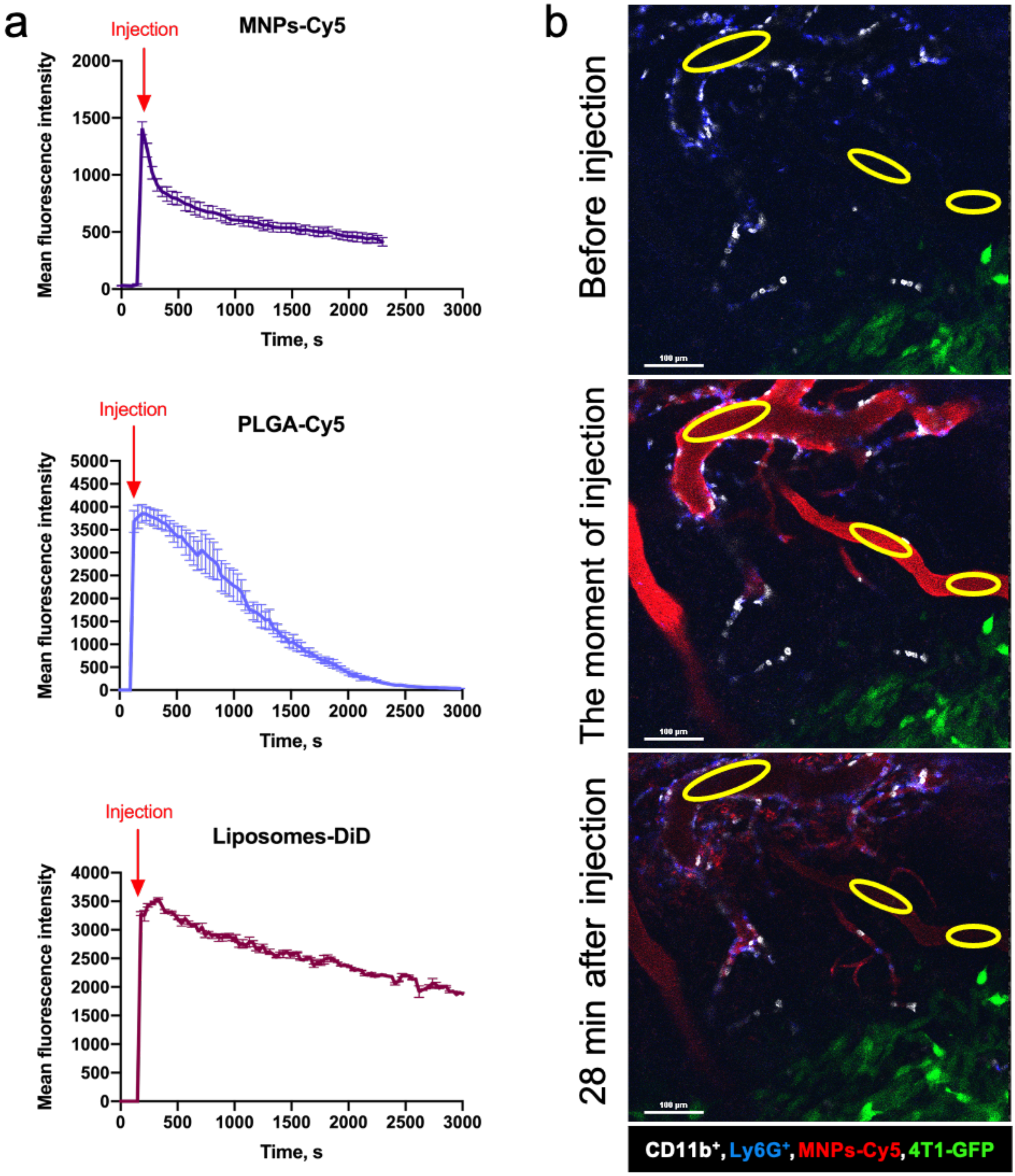

Figure S14. Nanoparticles' circulation time: (**a**) dynamics of Cy5/DiD mean fluorescence intensity in vessels' ROIs after NPs i.v. administration to 4T1-GFP tumor-bearing mice, results are shown as means ± SEM; (**b**) the example of Cy5 mean fluorescence intensity calculation in dynamics using ROIs after MNPs-Cy5 administration, IVM data post-processing.
